# Supplementary material for: Mathematical model for rod outer segment dynamics during retinal detachment
Source: PLoS One. 2024 Jun 7;19(6):e0297419. doi: 10.1371/journal.pone.0297419 (PMC11161088; doi:10.1371/journal.pone.0297419)
Supplement: S1 File — Here we show the criteria for unconditional ROS regeneration. (PDF) [file pone.0297419.s001.pdf]

Ranges for addition and removal rates that can lead to cell death

Criteria for unconditional ROS regeneration

Here we show additional calculations for how the conditions of the critical length L_C or ROS, found in the Section "Criteria for unconditional ROS regeneration" were determined. Retinal detachment leads to shortening of the ROS [s1] and eventual cell death for prolonged detachment [s2]. We assume that, for a rod cell in a detached retina whose outer segment (OS) is shortening, there exists a minimum length below which the rod cell is unable to survive, which we refer to as the critical length and denote by L_c. Here, we determine the range of values for the removal rate ($\gamma\alpha_s$) and the addition rate ($\delta\mu_0$) during RD that can drive the ROS below the critical length L_c, leading to cell death. For simplicity in notation, we denote the addition rate by $\nu = \delta\mu_0$ and the removal rate by $\varepsilon = \gamma\alpha_s$ and let $\hat{L}_T(t)$ be the length of the ROS at any time t. Then, by adding the L_g and L_m components of the feasible equilibrium point E_1 where

$$E_1 = \left(\gamma\bar{\alpha}_s\sqrt{\frac{\bar{\mu}_0\delta}{\gamma\bar{\alpha}_s(\gamma\bar{\alpha}_s+1)}},\quad \sqrt{\frac{\bar{\mu}_0\delta}{\gamma\bar{\alpha}_s(\gamma\bar{\alpha}_s+1)}},\quad \frac{\gamma\bar{\alpha}_s}{\bar{\mu}_s}\sqrt{\frac{\bar{\mu}_0\delta}{\gamma\bar{\alpha}_s(\gamma\bar{\alpha}_s+1)}}\right), \tag{1}$$

the total length of the ROS at equilibrium is given by

$$L_T^* = (\gamma\bar{\alpha}_s+1)\sqrt{\frac{\bar{\mu}_0\delta}{\gamma\bar{\alpha}_s(\gamma\bar{\alpha}_s+1)}} \tag{2}$$

where $L_T^* = \frac{\hat{L}_T}{L_{max}}$ is a dimensionless quantity representing the total length of the ROS. For cell death to occur, we must have this length fall below the critical value such that

$$\hat{L}_T < L_c \implies L_T^* < \frac{L_c}{L_{max}} \tag{3}$$

Substituting equation (2) into (3), squaring both sides and simplifying we arrive at

$$\bar{\mu}_0\delta(\gamma\bar{\alpha}_s+1) < \gamma\bar{\alpha}_s\frac{L_c^2}{L_{max}^2} \tag{4}$$

Recall from the Equilibrium and stability analysis Section that $\bar{\mu}_0 = \frac{\mu_0}{\alpha_g L_{max}}$ and $\bar{\alpha}_s = \frac{\alpha_s}{\alpha_g}$. Substituting these into inequality (4) and simplifying gives

$$\mu_0\delta(\gamma\alpha_s+\alpha_g) < \gamma\alpha_s\frac{\alpha_g L_c^2}{L_{max}}. \tag{5}$$

Denoting $\kappa = \frac{\alpha_g L_c^2}{L_{max}}$ in inequality (5), we get

$$\varepsilon(\nu-\kappa) < -\alpha_g\nu, \tag{6}$$

an inequality describing the relationship between disc addition and removal such that, if satisfied, will lead to rod cell death (i.e., total ROS length will eventually fall below the critical length L_c). Since $\varepsilon, \nu, \kappa, \alpha_g$ are all non-negative, inequality (6) holds only when

$$\nu < \kappa \quad \text{and} \quad \varepsilon > \frac{\alpha_g\nu}{\kappa-\nu} \tag{7}$$

Recall that Fig. 9 in the main text illustrates regions in $\nu\varepsilon$-plane where the ROS will eventually fall below the critical length L_c signifying rod cell death or can not fall below

L_c no matter how long the detachment lasts. In particular, if the rate of addition of new discs ($\nu = \delta\mu_0$) and removal of older discs ($\varepsilon = \gamma\alpha_s$) during RD satisfies the inequality in (7) (ie ν and ε falls within the unshaded portion of Fig. 9), the ROS will eventually decrease below the critical length (L_c) leading to cell death. On the other hand, if

$$\nu \geq \kappa \tag{8}$$

or

$$\nu < \kappa \quad \text{and} \quad \varepsilon \leq \frac{\alpha_g \nu}{\kappa - \nu} \tag{9}$$

the ROS cannot decrease below the critical length L_c. This means if the removal and addition rates of discs during RD fall within the shaded portion of Fig. 9, the rod cell cannot die no matter how long RD lasts.

[s1] Murakami Y, Notomi S, Hisatomi T, Nakazawa T, Ishibashi T, Miller JW, et al. Photoreceptor cell death and rescue in retinal detachment and degenerations. Progress in retinal and eye research. 2013;37:114–140.

[s2] Faude F, Francke M, Makarov F, Schuck J, G artner U, Reichelt W, et al. Experimental retinal detachment causes widespread and multilayered degeneration in rabbit retina. Journal of Neurocytology. 2002;30:379–390.
